# MZB1 at the ER-immunity interface: from antibody folding to disease vulnerability in autoimmunity, inflammation, and cancer

**DOI:** 10.7150/jca.125922

**Published:** 2026-02-11

**Authors:** Yu Zhang, Wei Yang, Xiaofang Yang, Yuqing Pan, Rong Guo, Dong Chen, Shicong Tang, Xi Zhang

**Affiliations:** 1 Department of Clinical Laboratory, the Third Affiliated Hospital of Kunming Medical University, Yunnan Cancer Hospital, Kunming 650118, Yunnan, China.; 2 Yunnan Key Laboratory of Laboratory Medicine, Kunming 650032, Yunnan, China.; 3 Department of Breast Surgery, the Third Affiliated Hospital of Kunming Medical University, Yunnan Cancer Hospital, Kunming 650118, Yunnan, China.; 4 Department of Ultrasound Department, the Third Affiliated Hospital of Kunming Medical University, Yunnan Cancer Hospital, Kunming 650118, Yunnan, China.

**Keywords:** MZB1, endoplasmic reticulum, immune homeostasis, molecular chaperone, tumor microenvironment, targeted therapy

## Abstract

MZB1 (marginal zone B and B1 cell-specific protein 1) is an endoplasmic reticulum-resident molecular chaperone that is predominantly expressed in marginal zone B cells, B1 cells, plasma cells, and pDCs. Functioning as a co-chaperone of GRP94 and BiP, MZB1 selectively facilitates the proper folding and secretion of immunoglobulin M (IgM) and J-chain-containing immunoglobulin A (IgA) dimers, and maintains the homeostasis of highly secretory cells by upregulating the expression of partner proteins such as BiP/GRP94. This review highlights the emerging role of MZB1 across various pathological conditions. In autoimmune diseases such as systemic lupus erythematosus (SLE) and rheumatoid arthritis (RA), MZB1 contributes to aberrant autoantibody and cytokine secretion. MZB1 also modulates inflammatory responses in conditions such as colitis and periodontitis by regulating the B cell-skewed inflammatory microenvironment. In oncology, MZB1 is overexpressed in breast cancer (BC), lymphoma, and multiple myeloma (MM), where it is associated with enhanced tumor cell proliferation and poor prognosis. Conversely, in hepatocellular carcinoma (HCC) and gastric cancer (GC), MZB1 is transcriptionally silenced due to promoter hypermethylation. In summary, the tissue-specific expression pattern of MZB1 endows it with potential as both a diagnostic biomarker and therapeutic target, holding significant implications for the exploration of future cancer prognosis and treatment strategies.

## 1. Introduction

The endoplasmic reticulum (ER)-resident protein MZB1 is a molecular chaperone encoded by a locus on chromosome 5q31.2. Its crystal structure displays a characteristic saposin-like fold and contains highly conserved cysteine residues as well as a C-terminal KDEL-like tetrapeptide motif that anchors it to the ER lumen. Due to these features, MZB1 is also referred to as CNPY5 or pERp1 1, 2. As the most abundantly expressed ER chaperone in marginal zone B cells, B1 cells, plasma cells, and pDCs(plasmacytoid dendritic cells) 3, MZB1 contributes to immune homeostasis through three primary mechanisms: (a) Promoting antibody maturation: as a co-chaperone of BiP/GRP94 (the ER-specific Hsp70/Hsp90 paralogs), it specifically aids in the oxidative folding and secretion of IgM and IgA (J-chain-containing dimer) 3-5; (b) Maintaining cellular signaling: It regulates B-cell integrin activation, calcium homeostasis, and the high-level secretion of interferon-alpha (IFN-α) by pDCs 6, 7; (c) Alleviating ER stress: By activating the ATF6 pathway, MZB1 upregulates chaperone protein expression, thereby supporting the survival of hypersecretory cells^6^.

Recent studies have revealed a paradoxical dual role of MZB1 in disease. On one hand, MZB1 exerts pro-pathogenic effects: it promotes excessive autoantibody and IFN-α production in SLE 6, 8; in RA, guanosine modification of MZB1 enhances autoantibody secretion 9; it is highly expressed in cancers such as breast cancer (BC), lymphomas, and multiple myeloma (MM), and is positively correlated with tumour progression 10-12. On the other hand, MZB1 also demonstrates protective functions: it helps maintain intestinal homeostasis in colitis through the IgA-J chain-mediated barrier 5; in hepatocellular carcinoma (HCC) and gastric cancer (GC), it is predominantly silenced via promoter hypermethylation, is associated with tumour suppression, and serves as an effective prognostic indicator 13, 14. These findings highlight the role of MZB1 as a key node linking endoplasmic reticulum quality control-immune response-disease pathology. However, its tissue-specific regulatory mechanisms (e.g., methylation silencing, post-translational modifications) and targeting strategies still need to be deeply explored. This review aims to systematically integrate the multidimensional roles of MZB1 in immune homeostasis, tumor microenvironment and disease, and provide theoretical basis for the development of novel diagnostic markers and targeted therapies.

## 2. Structure of MZB1

**Figure 1:**
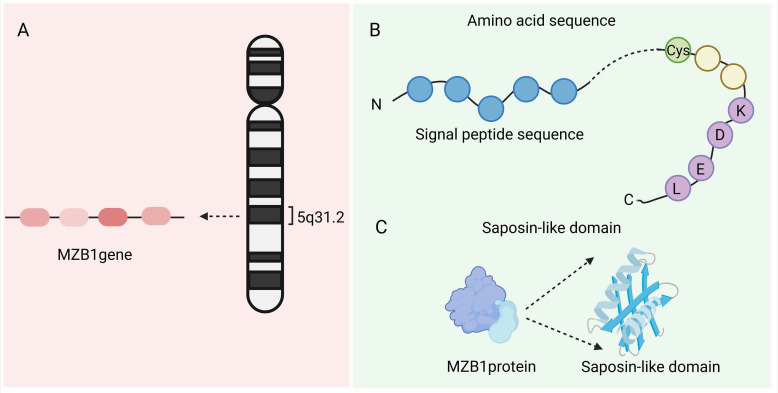
** Structure of MZB1.** A. The location of the MZB1 gene. B. The signal peptide, highly conserved cysteine residues, and C-terminal 'KDEL-like' tetrapeptide sequence of MZB1 protein. C. Saposin-like domain of MZB1 protein.

The MZB1 gene is located on chromosome 5q31.2 and contains four exons (NCBI Gene ID: 51237). It encodes an ER-resident protein with a molecular weight of approximately 18-19 kDa, comprising about 189 amino acids 1. The MZB1 protein (PDB ID:7AAH) includes a signal peptide (which directs the protein into the ER lumen), a highly conserved cysteine residue (which forms disulfide bonds), and a C-terminal “KDEL(Lys-Asp-Glu-Leu)-like” tetrapeptide sequence that facilitates ER retention. MZB1, also known as CNPY5, belongs to the ER-resident CNPY protein family and features a saposin-like domain, a structure commonly involved in lipid or protein binding 1. Crystal structure analysis confirms that MZB1 adopts a typical saposin fold with unique structural motifs, which collectively enable its function as a molecular chaperone in the ER. Owing to this role, it is also referred to as pERp1 (resident ER protein) 2. Under normal physiological conditions, MZB1 is primarily expressed in B-cell lineages, particularly in marginal zone B cells, B1 cells, and plasma cells. It is one of the most abundantly expressed ER proteins in these cell types, highlighting its specialized role in supporting their secretory functions 3 (Figure 1).

## 3. Role of MZB1 in the immune system

**Figure 2:**
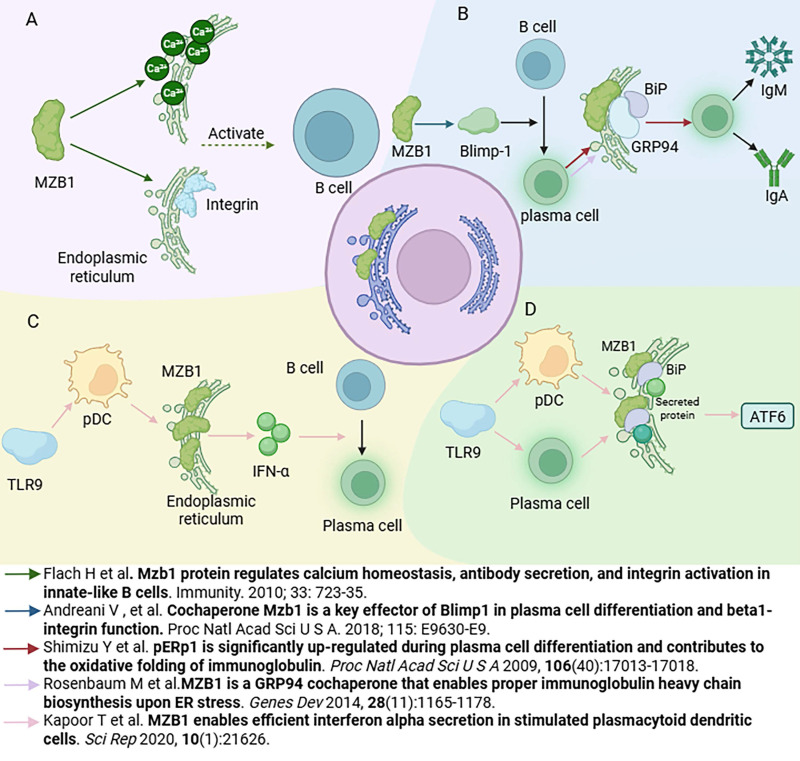
** Role of MZB1 in the immune system.** A. The role of MZB1 in B cell development and function. B. The role of MZB1 in plasma cell differentiation and antibody secretion. C. The effect of MZB1 on the secretory function of plasmacytoid dendritic cells. D. The effect of MZB1 on the endoplasmic reticulum stress response.

MZB1 plays a critical and multifaceted role in the immune system, functioning as a folding cofactor and quality control mediator for antibodies and integrins within the endoplasmic reticulum. Through its specialized structural domains and chaperone activity, MZB1 supports the proper assembly of immune molecules, maintains calcium homeostasis, and alleviates ER stress to preserve cellular balance 4, 6, 7, 15 (Figure 2).

### 3.1. B cell development and function

MZB1 is highly expressed in innate-like B cell subsets, particularly marginal zone B cells and B1 cells, and plays a crucial role in their development and function 3. Marginal zone B cells and B1 cells are cell populations that mount a rapid antibody response to pathogens. Marc Rosenbaum *et al.* showed that MZB1 deficiency impairs the marginal zone B cell-mediated humoral immune response, in particular on early IgM antibody production to B cell-independent antigens 4. MZB1 regulates Ca^2+^ homeostasis and endoplasmic reticulum Ca^2+^ reserves. In addition, MZB1 may influence integrin signalling by modulating the redox enzyme ERp57. Integrin primarily is involved in the adhesion process of marginal zone B cells to integrin ligands VCAM-1 or ICAM-1 upon chemokine induction.^16^. In MZB1-deficient mice, B1 cells exhibit reduced B cell receptor (BCR)-induced calcium mobilization and impaired β1 integrin-dependent adhesion capacity 7. Thus, MZB1 contributes to the regulation of calcium signaling and adhesion molecule function in B cells, thereby facilitating B cell activation, adhesion, and migration.

### 3.2. Plasma cell differentiation and antibody secretion

The transcription factor Blimp-1 is a master regulator of plasma cell differentiation, and MZB1 acts as a key downstream effector in the Blimp-1 signaling pathway during the transition from B cells to plasma cells 15. MZB1 is essential for the efficient production of antibodies by plasma cells 9. Its role in IgM secretion has been confirmed in primary B cells: MZB1 downregulation impairs IgM secretion, while its overexpression enhances IgM production in marginal zone B (MZB) cells and follicular B (FoB) cells upon lipopolysaccharide (LPS) stimulation 7. The proper folding and secretion of antibodies in the endoplasmic reticulum rely on the ER-resident chaperones BiP and GRP94, which ensure protein quality control and homeostasis 17. MZB1 is a chaperone protein of GRP94/BiP.Yuichiro Shimizu's group demonstrated that MZB1 interacts with the BiP complex to facilitate oxidative folding of immunoglobulin domains 3. Moreover, MZB1 forms a complex with GRP94 and acts as its co-chaperone, enhancing the association between GRP94 and the immunoglobulin μ heavy chain, thereby improving the efficiency of IgM antibody assembly and secretion 4, 18. Similarly, MZB1 promotes the efficient folding and secretion of IgA antibodies, but has minimal impact on IgG production 5, 19. In addition, MZB1 deficiency does not significantly affect the early stages of plasma cell differentiation, as expression of transcriptional regulators such as Blimp-1 and XBP1 remains unchanged. However, it compromises the secretory efficiency of plasma cells in the later differentiation stages 4. Thus, MZB1 primarily functions during the effector phase of plasma cells by enhancing the folding and secretion of IgM and IgA antibodies, thereby expanding their secretory capacity.

### 3.3. Role of pDCs

In addition to B cells, MZB1 mRNA is also expressed in pDCs 20. Although pDCs exhibit a lymphoid morphology at rest, they acquire dendritic features upon activation, hence the name “plasmacytoid dendritic cells” 21. Tanya Kapoor *et al.* reported that MZB1 expression levels in murine pDCs are comparable to those in marginal zone B cells and higher than in follicular B cells 6. Functionally, MZB1-deficient pDC were significantly impaired in their ability to secrete IFN-α in response to TLR9 stimulation, but had little effect on the small amount of IFN-α produced by low levels of stimulation (e.g., weak TLR7 stimulation), suggesting that MZB1 functions primarily under conditions of high-intensity secretion 6. When large amounts of IFN-α are synthesized, MZB1 facilitates its proper folding and transport; in the absence of MZB1, IFN-α accumulates in the endoplasmic reticulum and fails to be efficiently secreted. In addition, pDC can promote the differentiation of B cells into plasma cells by secreting IFN-α 6. Co-culture experiments showed that wild-type pDC significantly promoted B cell differentiation to CD138^+^ plasma cells and IgM production, whereas this function was significantly impaired in MZB1-deficient pDC. Supplementation with exogenous IFN-α or blockade of the IFN receptor restored or impaired the differentiation effect, respectively, suggesting that MZB1 deficiency leads to insufficient secretion of IFN-α by pDC to efficiently assist B cell differentiation 6. In conclusion, MZB1 can stimulate pDC to secrete IFN-α. Moreover,given that pDCs are a major source of IFN-α in autoimmune diseases such as SLE 22, 23, MZB1 may serve as an important molecular link between innate and adaptive immunity.

### 3.4. Endoplasmic reticulum stress response

Plasma cells and pDC, which have the highest secretory load among hematopoietic cells, require robust ER protein folding, which involves induction of ER stress, increased expression of protein folding chaperones, and expansion of the ER 24, 25. The accumulation of unfolded proteins in the ER triggers ER stress, which is alleviated through activation of the unfolded protein response (UPR), thereby restoring protein homeostasis. The UPR consists of three canonical signaling pathways: inositol-requiring enzyme 1 (IRE1), PKR-like ER kinase (PERK), and activating transcription factor 6 (ATF6) 26, 27. MZB1 functions as a molecular "lubricant" within the UPR machinery, supporting highly secretory cells in managing their protein folding demands. However, the relationship between MZB1 and IRE1 / PERK is not clear. In particular, MZB1 activates the ATF6 pathway: under TLR9-mediated stimulation in plasma cells and pDCs, MZB1 enhances BiP-mediated binding of secretory proteins, promoting early activation of ATF6. This leads to upregulation of chaperone expression (e.g., BiP and GRP94) and facilitates ER expansion, thus helping the cell adapt to high secretory demands 6. These findings suggest that MZB1, by indirectly modulating the UPR, enables secretory cells to resolve ER stress and maintain homeostasis under strong stimulation. In summary, MZB1 serves as a critical molecular link between the immunosecretory function and the ER stress response in highly secretory cells such as B cells and pDCs. On one hand, it ensures the efficient folding and secretion of large quantities of antibodies and cytokines; on the other, it activates protective UPR mechanisms to prevent apoptosis during peak secretory activity.

## 4. The relationship between MZB1 and immune-related diseases and its research progress

**Figure 3:**
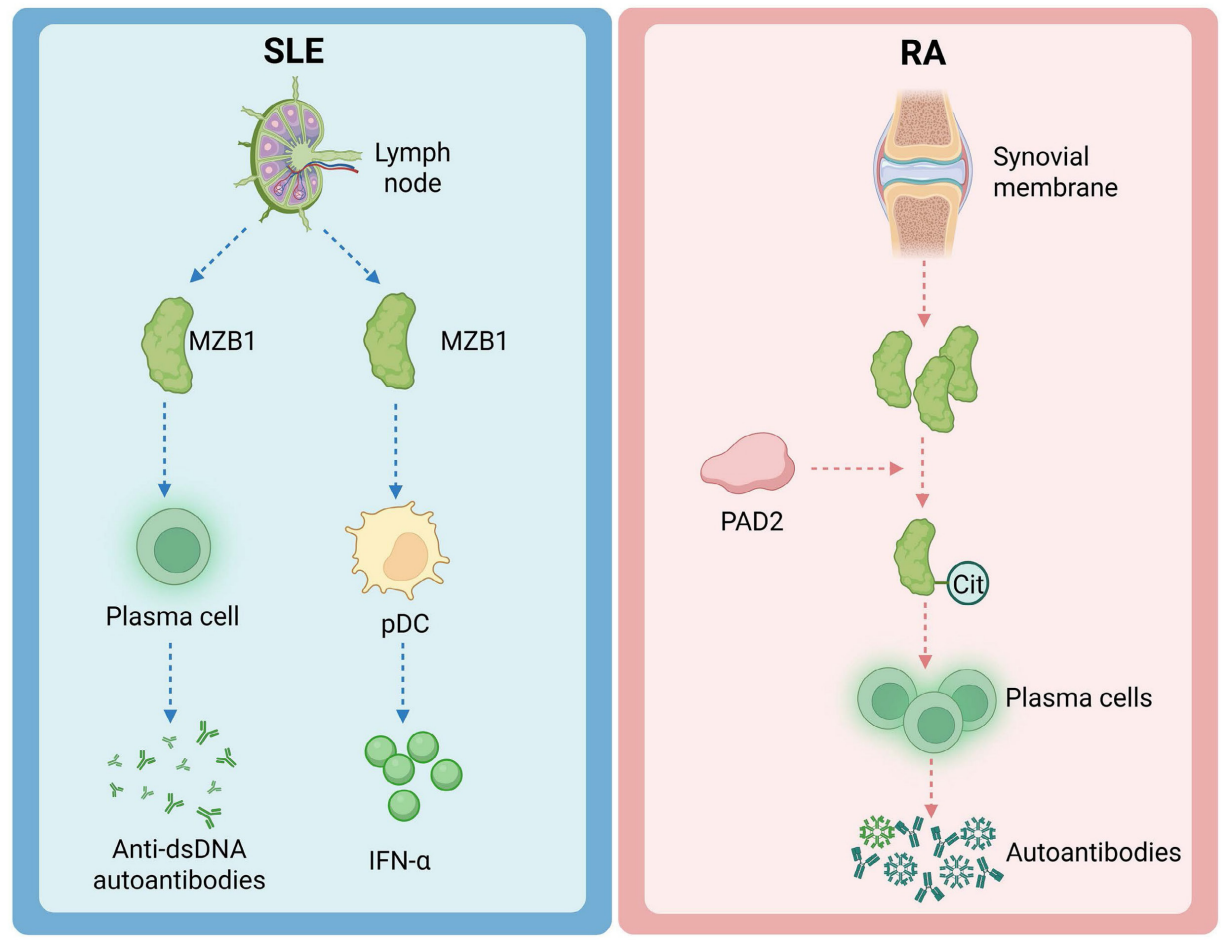
** The effects of MZB1 on SLE and RA**.

MZB1 is also important in a variety of autoimmune and immune-related diseases. Such diseases are often accompanied by abnormal humoral immune responses involving dysregulation of plasma cells and antibody production. SLE and RA are discussed below as representatives (Figure 3).

### 4.1. Systemic lupus erythematosus

SLE patients are characterized by B-cell hyperfunction and overproduction of cytokines IFN-α, TNF and IL-1 28. A study conducted proteomic analysis of lymph node biopsies from SLE patients identified MZB1 as one of the proteins differentially upregulated in lymphoid tissues of SLE patients 8. Further immunohistochemical validation showed that MZB1 was abundantly located in the interfollicular zone and scattered in the germinal centers in lymph nodes of SLE patients, in association with CD138⁺ plasma cells and IRTA1⁺ marginal zone B cells 8. Correspondingly, a similar phenomenon was observed in a murine model of spontaneous lupus, with elevated levels of MZB1 in splenic marginal zone B cells and plasma cells in aged rats 8. Functional experiments suggest that MZB1 is closely related to the pathogenesis of lupus: treatment of lupus mice with endoplasmic reticulum stress inducers selectively induced apoptosis in plasma cells with high expression of MZB1, which resulted in a significant decrease in serum anti-dsDNA autoantibodies of the mice, which suggests that high titers of autoantibodies in the condition of lupus depend partly on plasma cells with high expression of MZB1 8. In addition, as mentioned previously, pDC are the main source of IFN-α in lupus patients 29, and high MZB1 expression can fuel pDC to secrete large amounts of IFN-α 6. Collectively, these findings indicate that MZB1 is upregulated in two key pathogenic pathways in SLE—B cells and pDCs: on one hand, it supports the production of pathogenic autoantibodies by plasma cells, and on the other, it amplifies IFN-α-mediated immune responses via pDCs 6, 8. Therefore, MZB1 has been proposed as a potential therapeutic target in SLE, with the aim of selectively depleting antibody-overproducing plasma cells 8. Although this strategy remains in the early conceptual stage, it highlights the central role of MZB1 in SLE pathogenesis and its therapeutic relevance.

### 4.2. Rheumatoid arthritis

RA is a chronic inflammatory disease characterized by the presence of autoantibodies, such as rheumatoid factor (RF) and anti-cyclic citrullinated peptide (anti-CCP) antibodies 30. Plasma cells that produce large quantities of autoantibodies are commonly found in RA patients, and these cells can form ectopic lymphoid structures within synovial tissues. Notably, MZB1 is highly expressed in plasma cells within RA synovium 8. In addition, an interesting mechanism comes from studies of arthritis-associated lung disease: RA patients often suffer from interstitial inflammation of the lungs, where high expression of citrullination enzyme 2 (PAD2) in the lungs is able to citrullinate the MZB1 protein 9. Citrullination, a post-translational modification that converts arginine residues into citrulline, is strongly associated with the generation of anti-citrullinated protein/peptide antibodies (ACPAs) in RA pathogenesis 31. Lee *et al.* identified MZB1 as one of the citrullinated proteins enriched in lung tissues of RA patients, and demonstrated that PAD2-mediated citrullination of MZB1 enhances the ability of plasma cells to secrete IgM and IgA antibodies 31. This partly explains why RA patients produce excessive amounts of IgM-RF and IgA-like autoantibodies 32. Beyond plasma cells, MZB1 has recently been reported to be highly upregulated in T cells and monocytes within leukocyte-rich RA synovial tissues 33. MZB1 is also being explored as a potential biomarker to distinguish RA from spondyloarthritis (SpA) 34. Additionally, recent studies suggest that MZB1 plays a role in B-cell maturation and antibody generation, and is associated with the development of anti-drug antibodies (ADAs), which may impact therapeutic efficacy in RA patients 35. Thus, it is clear that MZB1 is not only increased in expression in RA, but also becomes part of the immune aberrant loop specific to RA. In the future, it is expected that MZB1 will be used as a node to develop novel therapies for RA, such as utilizing the role of MZB1 in regulating autoantibody production to alleviate the disease.

## 5. Relationship between MZB1 and inflammatory diseases and its research progress

As an important mediator involved in the correct folding of antibodies in the ER, MZB1 is often upregulated in humoral immunity. However, its role may vary in different inflammatory conditions, which might be due to the fact that it mainly depends on the different internal environment (Table 1).

### 5.1. Periodontitis

Periodontitis is a common chronic inflammatory disease that affects the supporting structures of the teeth, leading to progressive destruction of periodontal tissues and alveolar bone, which can ultimately result in tooth loss 36. In the context of periodontitis, MZB1 may act as part of the local humoral immune response, regulating the synthesis of immune proteins and the differentiation of immune cells 37. A microarray-based gene expression profiling study of aggressive periodontitis revealed that MZB1 is significantly upregulated in gingival tissues of affected patients 38. Similarly, Esra Guzeldemir-Akcakanat *et al.* identified MZB1 as a signature molecule of chronic periodontitis through genome-wide transcriptomic and proteomic analyses 39. These findings were further supported by transcriptome sequencing and histological studies, which consistently demonstrated elevated MZB1 expression in inflamed gingival tissues, providing new insights into the genetic drivers of periodontal inflammation 40, 41. A recent study also reported changes in the composition of immune cells between healthy and diseased gingival tissues, including differences in B cells, T cells, dendritic cells, and neutrophils, with MZB1 expression highly correlated with B cell infiltration 42. Furthermore, MZB1 expression was found to increase during T cell differentiation 43, suggesting broader immune regulatory roles. Mechanistically, MZB1 has been identified as a direct target of miR-185-5p, and it was shown to inhibit human periodontal ligament cell migration via the NF-κB signaling pathway. This may represent a key regulatory mechanism by which MZB1 modulates periodontal inflammation and tissue remodeling 44. Although the current understanding of MZB1's molecular mechanisms in periodontitis remains limited, it presents a promising avenue for future research and may emerge as a novel target in periodontitis-related diagnostics or therapeutics.

### 5.2. Colorectal inflammation

Chronic inflammation is known to be associated with the development of many types of cancer, and intestinal inflammation may induce oncogenic mutations and promote colorectal cancer 45. IgA is the most abundant antibody in mucosal secretions, and plays an essential role in intestinal homeostasis regulation and mucosal immunity 46. Dimeric IgA molecules are covalently linked via a joining J chain, which is essential for their translocation across epithelial barriers to mucosal surfaces 47. Ermeng Xiong *et al.* found that MZB1-deficient mice have an increased susceptibility to dextran sulfate sodium (DSS)-induced colitis, and that IgA and IgM levels were elevated in the intestines of MZB1-overexpressing mice after DSS administration. In contrast, IgA levels did not increase in MZB1-deficient mice, while IgM levels increased only slightly. Exogenous addition of IgA restored the normal sensitivity of MZB1-deficient mice to DSS-induced colitis 5. Mechanistically, MZB1 deficiency impairs IgA secretion by plasma cells and disrupts J chain—IgA association, thereby compromising the efficient transport of IgA to the intestinal mucosa. These findings provide reverse validation that MZB1 promotes IgA secretion and plays a protective role in suppressing the onset and progression of colitis 5. In addition, a research team found that MZB1 was significantly overexpressed in ulcerative colitis by whole blood RNA sequencing. However, there is currently little relevant research available, and the specific role of MZB1 in the development of colitis requires further investigation 48.

### 5.3. Other inflammatory diseases

In addition to its roles in periodontitis and colitis, MZB1 is also expressed and functionally relevant in a range of other inflammatory conditions. For example, Kyoko Oshina *et al.* reported via RNA sequencing that MZB1 is among the upregulated genes in the endometrium of patients with chronic endometritis 49. In type 2 chronic rhinosinusitis with nasal polyps (CRSwNP), the ER stress level of B cells was significantly elevated, and MZB1 showed co-localization with plasma cells and mature B cells and was significantly upregulated. Further experiments revealed that MZB1 stimulated upregulation of ER stress markers and IgE mRNA expression 50. In addition, a recent study on MZB1 and acute pancreatitis (AP) used bioinformatic analysis and *in vitro* cell experiments to demonstrate that MZB1 expression levels are significantly elevated in AP cells and tissues. Furthermore, MZB1 overexpression can promote pancreatic cell proliferation and inhibit cell apoptosis, mitochondrial dysfunction, and inflammation by regulating the PI3K-AKT signaling pathway, thereby playing a key role in AP 51. The possible reason for the up-regulation of MZB1 in inflammatory diseases is that MZB1 is expressed in B-cells and correlates with the secretion of immunoglobulins IgM and IgA, which also provides important clues for the study of MZB1 and inflammatory diseases.

## 6. MZB1 expression and role in tumors

**Figure 4:**
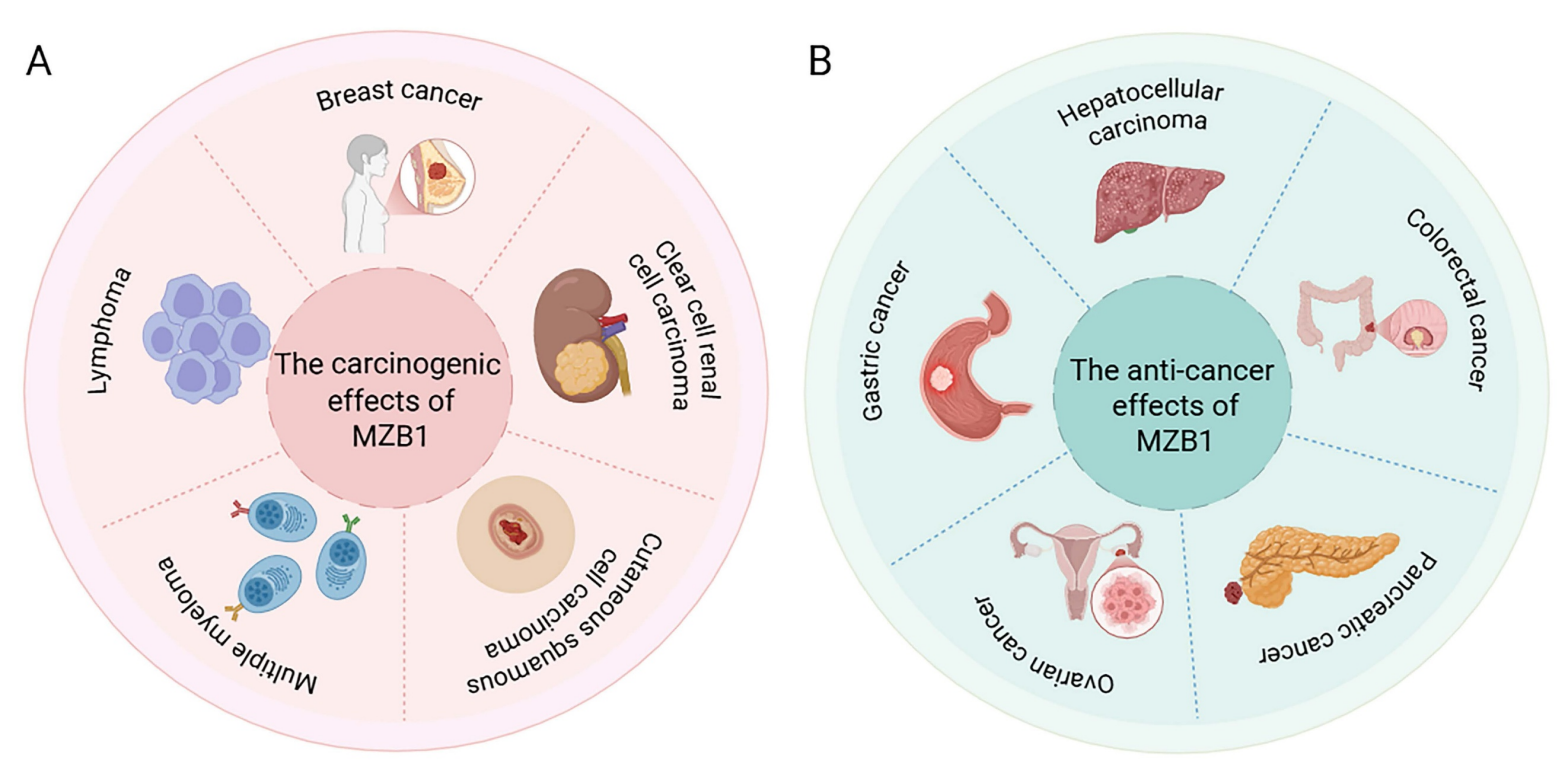
** Expression and function of MZB1 in tumours.** A. The carcinogenic effects of MZB1. B. The anti-cancer effects of MZB1.

MZB1 is abnormally expressed in a variety of tumors, which is correlated with the differentiation status of tumor cells, the immune microenvironment and disease prognosis. Tissue-specific secretory demands and UPR dependency may select for high expression of MZB1 (e.g., plasma-cell tumors, ER^+^ BC), whereas tumors with methylation drift may silence MZB1, losing ER-stress checkpoints. The following summarizes the research progress of MZB1 in several types of typical cancers (Figure 4 and Table 2).

### 6.1. Breast cancer

Recent studies have found that MZB1 is highly expressed in BC by bioinformatics analysis 52 and is associated with BC prognosis 53. Similarly, a study using eXplainable Artificial Intelligence (XAI) found that MZB1 could be used as an early, non-invasive diagnostic and prognostic biomarker for BC 54. Manabu Watanabe's team found that MZB1 expression was strongly correlated with hormone receptor status in BC, and they examined multiple mammary cell lines, which were detected mRNA expression of MZB1 only for estrogen receptor (ER⁺) breast cancer cells, whereas ER-negative cell lines and normal mammary epithelial cells barely expressed MZB1 10. Among ER⁺ BC patients, high MZB1 expression was associated with worse clinical outcomes: disease-free survival (DFS) was significantly shorter in MZB1-positive patients, and multivariate analyses identified MZB1 positivity as an independent prognostic factor for poor outcome 10. The specific mechanism may involve heightened activity within the endoplasmic reticulum and protein synthesis pathways of ER⁺ breast cancer cells. Upregulation of MZB1 may confer enhanced protein folding and secretion capabilities to these cells, thereby promoting tumour cell survival and invasion 10. However, this potential mechanism requires extensive validation through both *in vivo* and *in vitro* studies. In summary, MZB1 expression correlates with breast cancer progression, particularly within the hormone receptor-positive subtype.

### 6.2. Lymphoma

MZB1 is frequently overexpressed in B cell derived malignancies, including chronic lymphocytic leukemia (CLL), follicular lymphoma (FL), and diffuse large B-cell lymphoma (DLBCL), and its expression positively correlates with disease aggressiveness 11. Herold *et al.* analyzed the gene expression profiles of peripheral blood tumor cells in patients with CLL, and initially found that high expression of MZB1 was associated with poor prognosis. This was subsequently validated in several independent cohorts, and in 139 CLL patients, both overall and progression-free survival were significantly shorter in the MZB1 mRNA high-expression group 11. Similarly, publicly available datasets for FL and DLBCL revealed that elevated MZB1 expression was associated with worse clinical outcomes. Notably, in DLBCL, the prognostic significance of MZB1 remained consistent across different molecular subtypes, including both the activated B cell like (ABC) and germinal center B cell like (GCB) subtypes, indicating that MZB1 functions as an additional adverse prognostic factor regardless of molecular classification 11. Given the critical role of MZB1 in B cell function, its overexpression may enhance the antibody-secreting capacity of tumor B cells, thereby promoting tumor cell survival 11. Although MZB1 exhibits high expression levels across multiple study cohorts, this remains dependent on subtype and plasma cell characteristics.

### 6.3. Multiple myeloma

MM is a malignant proliferative disease of plasma cells, and MZB1 is highly expressed in MM and plays a tumor-promoting role 55. Chanukuppa *et al.* conducted proteomic analyses and found that MZB1 protein levels were significantly elevated in bone marrow mononuclear cells from MM patients compared to healthy controls 12. In the multiple myeloma cell line RPMI-8226, MZB1 knockdown via shRNA led to marked phenotypic changes: proliferation was slowed, cell cycle arrest was induced, and apoptosis was significantly increased. Soft agar colony formation assays further showed that MZB1-deficient myeloma cells had reduced anchorage-independent growth, indicating impaired tumorigenic potential 12. Mechanistically, MZB1 knockdown resulted in impaired multiple proliferation-related signals: the phosphorylation levels of the key pro-survival pathways AKT and ERK were significantly reduced, and the expression of cyclins Cyclin D1, Cyclin A, and Cyclin B, which are required for the cell cycle, was down-regulated 12. Additionally, the study suggested that MZB1 may interact with the immunoglobulin J chain to cooperatively promote the malignant phenotype of myeloma cells 12. Emerging evidence also indicates that MZB1 is an aberrantly spliced gene in MM, and disruption of normal RNA splicing patterns is increasingly recognized as a driver of tumorigenesis 56. Although current data remain limited, these findings collectively suggest that MZB1 functions as an oncogene in multiple myeloma by sustaining tumor cell proliferation and survival.

### 6.4. Hepatocellular carcinoma

In contrast to the cancer types discussed above, MZB1 is expressed at low levels in HCC. Matsumura *et al.* identified frequent MZB1 silencing in HCC by integrating DNA methylation microarray and gene expression profiling data, and found that hypermethylation of the MZB1 promoter region was a major mechanism contributing to its transcriptional suppression 13. In a cohort of 162 patients with primary HCC, immunohistochemical analysis revealed that MZB1 protein expression was significantly reduced in tumor tissues, and Kaplan-Meier survival analysis showed that loss of MZB1 expression was associated with poorer prognosis 13. Furthermore, in hepatocellular carcinoma cell lines with low MZB1 expression, extracellular transfection of MZB1 inhibited tumor cell proliferation and induced G1-phase cell cycle arrest 13. Animal experiments have also shown that restoring MZB1 expression can slow tumor growth 13. It is therefore inferred that MZB1 is associated with HCC, and its loss promotes the proliferation of hepatocellular carcinoma cells, thereby facilitating carcinogenesis. Given that MZB1 silencing is primarily driven by promoter CpG island hypermethylation, it may serve as a methylation-based biomarker or therapeutic target, particularly in the context of epigenetic therapies such as demethylation agents.

### 6.5. Gastric cancer

In GC, early microarray analysis found that MZB1 (formerly known as MGC29506) was downregulated in intestinal-type GC tissues, suggesting an association between MZB1 and GC development 57. Lin Xia *et al.* found that the expression of MZB1 in GC tissues was lower than that in samples from adjacent non-tumor tissues by immunohistochemistry 58. In addition, functional assays showed that GC cells induced by MZB1 gene transfection were arrested in the G0/G1 and S phases of the cell cycle and MZB1 inhibited the proliferation of GC cells 58. Similarly, Mitsuro Kanda *et al.* showed by transcriptome analysis that the expression level of MZB1 was significantly reduced in primary GC tissues compared to the corresponding normal gastric mucosa, and that MZB1 knockdown increased the proliferation, invasion, and migration of the GC cells, further suggesting that MZB1 may act as a novel suppressor gene in GC^14^.

### 6.6. Other solid tumors

In addition to the cancer types discussed above, several studies have provided preliminary evidence for the role of MZB1 in other solid tumors. In cutaneous squamous cell carcinoma (cSCC), MZB1 may co-express with the lncRNA-mRNA network and may be associated with tumour progression 59. MZB1 expression is upregulated in clear cell renal cell carcinoma (ccRCC) and is associated with poor prognosis in ccRCC patients 60. Conversely, Huiya Xu *et al.* determined through proteomic analysis that MZB1 exhibits a negative correlation with the clinical staging of mucoid tubular and spindle cell carcinoma (MTSCC) 61. In addition, MZB1 may be an important hub gene in lung non-small cell lung cancer (NSCLC) tissues 62. Moreover, there is a positive correlation between MZB1 and PD-L1 in lung squamous cell carcinoma 63. Xiaohong Zhu *et al.* demonstrated that overexpression of MZB1 could counteract the inhibitory effects of miR-103-3p on the migration and metastasis of NSCLC cell lines 64. Finally, several studies based on bioinformatics analyses and functional experiments have reported that high MZB1 expression is positively associated with prognosis in cancers such as pancreatic cancer 65, 66, ovarian cancer 67, and colorectal cancer 68, 69. However, the specific molecular mechanisms behind these correlations have not been fully elucidated through experiments. Further validation can be achieved through knockdown and backfilling experiments using animal and cell models.

## 7. Discussion

In summary, the ER protein encoded by the MZB1 gene has important functions in the immune system at multiple levels. From a basic research perspective, MZB1 is a chaperone protein essential for antibody-secreting cells (plasma cells and some innate immune cells) to adapt to high secretory loads, regulating the assembly and folding of antibodies and cytokines as well as the ER stress response 6. Through interactions with ER chaperones such as GRP94, MZB1 promotes the maturation of immunoglobulins IgM and IgA, as well as specific receptor proteins such as Toll-like receptors (TLRs) and integrins. In addition, it contributes to the maintenance of intracellular calcium homeostasis and integrin-mediated adhesion signaling 2, 4, 7. In immune organs, MZB1 is highly expressed in marginal zone B cells, B1 cells, and plasma cells, contributing to rapid natural antibody response and high-affinity antibody production 70. In pDC, MZB1 similarly safeguards the high secretion of IFN-α 6. Together, these findings highlight MZB1 as a key “quality control” regulator of the humoral immune response, whose functional significance has only recently begun to receive widespread attention.

Clinical studies have shown that MZB1 is aberrantly expressed in a variety of disease states and becomes a “player” in disease development. MZB1's impact depends on whether the tissue context contains antibody-secreting programs (plasma cells/pDC secretory stress), epithelial tumor epigenetic state, and whether disease is driven by pathogenic humoral output (autoantibodies, IFN-I) and barrier-protective IgA. In autoimmune diseases such as SLE and RA, MZB1 is upregulated throughout the pathogenesis: in SLE, MZB1 promotes a dual excess of autoantibodies and inflammatory factors 6, 8, while in RA, post-translational modification of MZB1 exacerbates abnormal plasma cell activation 9. These findings suggest that MZB1 may be an enabler of autoimmune responses, and inhibition of MZB1 expression is expected to alleviate the pathologic process of related diseases 8. In inflammatory diseases, MZB1 plays multiple roles. For instance, MZB1 drives chronic inflammation in periodontitis, affecting the synthesis of immune proteins and the maturation of immune cells. It may play the role of a pro-inflammatory cytokine in hormone regulation, but this inference requires extensive experimental verification 37. In colorectitis, high MZB1 expression promotes IgA secretion from plasma cells and enhances J chain-IgA association, which decreases susceptibility to colitis 5. In cancer, MZB1 serves as a poor prognostic factor. Its elevated expression in certain solid tumours (such as breast cancer) correlates with tumour progression and adverse outcomes 10. On the other hand, in certain tumours (such as HCC), it functions as a tumour suppressor gene, silenced by methylation, and its deletion is associated with tumour progression 13. In B cell derived hematological malignancies, MZB1 is universally upregulated and associated with an aggressive disease phenotype, suggesting a dependency of malignant B cells on MZB1 11. Particularly in plasma cell tumors (myeloma), MZB1 has been shown to be a key protein in maintaining tumor proliferation and could be a potential therapeutic target for future MM 12. These studies provide powerful clues for future studies exploring molecular biomarkers related to tumor prognosis and targeted therapy.

Future research on MZB1 will be divided into several directions:(1) Mechanistic level: to deeply analyze the molecular mechanism of MZB1 in regulating protein folding, for example, how its saposin domain interacts with immunoglobulin heavy chain, BiP/GRP94, and how modifications such as citrullination affect its function; (2) Disease models: construct mouse models with high expression or deficiency of MZB1, and observe phenotypic changes in autoimmune diseases, inflammatory diseases, and tumor models to clarify the causal role of MZB1 in pathological processes; (3) Clinical application: develop interventions targeting the MZB1 pathway. For example, small molecules or antibodies are used to block the action of MZB1 and its chaperone proteins in order to selectively inhibit disease-causing plasma cells in autoimmunity; and in tumors, treatment strategies such as activating the silenced MZB1 gene (e.g., with demethylating drugs) or targeting and eliminating MZB1-high tumor cells (e.g., with MZB1-specific T cell immunotherapy) are being explored. With the expansion of the functional spectrum of MZB1, this molecule has the potential to move from basic research to clinical translation, becoming a new focal point connecting immunology and oncology. In conclusion, research on the structure and function of the MZB1 gene provides important clues for understanding the coupling between endoplasmic reticulum homeostasis and immune function, and its roles in diseases warrant further exploration and utilization.

## Figures and Tables

**Table 1:** The relationship between MZB1 and inflammatory diseases and its research progress

Disease type	Changes in MZB1 expression	Key Functions/Mechanisms	Species	Research methodology
Periodontitis	Significantly upregulated in gingival tissues	Drives chronic inflammation 39 and affects immune protein synthesis 37 and B cell infiltration 42. Inhibits periodontal ligament cell migration through the NF-κB pathway 44	Human, mouse	RT-qPCR, Western blotting, Microarray, Transcriptomics, Histologic analysis.
Colorectitis	Overexpressed in ulcerative colitis	Promotes IgA secretion from plasma cells and maintains intestinal mucosal immunity MZB1 deficiency results in decreased IgA and exacerbates DSS-induced colitis 5	Mouse	Flow Cytometry, Western blotting, Mouse model, RNA sequencing
Chronic endometritis	Upregulated in the endometrium	Involved in disease progression as an inflammation-associated gene, exact mechanism not defined 49.	Human	Immunohistochemistry, RNA sequencing, RT-qPCR,
Type 2 chronic rhinosinusitis with nasal polyps	Significantly upregulated in B cells	Upregulated ER stress markers and IgE expression 50.	Human	Western blotting, RNA sequencing, Immunohistochemistry
Acute pancreatitis	Elevated in pancreatic tissues and cells	Promotes pancreatic cell proliferation and inhibits apoptosis and mitochondrial dysfunction through the PI3K-AKT pathway 51.	Mouse	RT-qPCR, Western blotting, Mouse model, *In vitro* experiments

**Table 2:** The expression of MZB1 in cancer and its prognosis

Type of cancer	MZB1 expression	Correlation study	Species sample types and quantities, database	Prognosis	Reference documentation
Breast cancer (BC)	Up-regulation	RT-qPCR, Immunohistochemistry, Single-cell RNA-sequencing	13 BC cell lines and two non-cancerous mammary cell lines, BC patients (n=114) and non-cancerous specimens, and the cancer genome atlas (TCGA) for breast cancer	Related to poor prognosis of breast cancer patients	10,52,53
Lymphoma	Up-regulation	RT-qPCR, Microarray data	139 CLL patients	MZB1 is a prognostic marker for untreated and relapsed CLL, FL, and DLBCL	11
Multiple myeloma (MM)	Up-regulation	RT-qPCR, Western blotting, Proteomics analysis	32 patients with multiple myeloma and 32 patients with non-hematologic malignancies	MZB1 can accelerate the cycle progression of MM plasma cells and lead to malignant development to some extent.	12
Hepatocellular carcinoma (HCC)	Low expression	RT-qPCR, Immunohistochemistry, Western blotting, Whole genome methylation screening, Methylation analysis	162 cases of primary HCC, 15 HCC cell lines and 2 hepatoblastoma cell lines	MZB1 protein silencing is significantly and independently associated with poor prognosis.	13
Gastric cancer (GC)	Low expression	RT-qPCR, Immunohistochemistry, Transcriptome analysis, PCR array analysis, Methylation analysis	Primary gastric cancer and adjacent non-cancerous tissues of 200 patients, 11 cell types and 94 cases of patients with stage II/III gastric cancer who received radical surgery	The decreased expression of MZB1 is an independent predictor of recurrence in patients with stage II / III GC	14
Clear cell renal cell carcinoma(ccRCC)	Up-regulation	Western blotting, Immunohistochemistry, Single-cell sequencing data	59 pairs of human ccRCC and adjacent normal tissues	MZB1 is associated with poor prognosis in ccRCC patients	60
Mucinous tubular cell carcinoma (MTSCC)	Up-regulation	Proteomics spectrum analysis, Immunohistochemistry, Immunofluorescence	32 cases of MTSCC and 36 cases of type 1 papillary renal cell carcinoma (PRCC)	The expression of MZB1 in MTSCC is negatively correlated with the clinical stage of the tumor	61
Non-small cell lung cancer (NSCLC)	Up-regulation	Western blotting, RT-qPCR	12 tumor samples from NSCLC patients and their corresponding adjacent tissues	MZB1 is a potential prognostic biomarker for NSCLC	64
Pancreatic cancer	Up-regulation	Proteomics analysis, Bioinformatics analysis	72 patients with resectable PDAC, 3 pairs of high CD8 TIL and low CD8 TIL, and the TCGA data base	High expression of MZB1 correlates with disease-free and overall survival	65,66
Ovarian cancer	Low expression	Bioinformatics analysis	TCGA data base	High expression of MZB1 is positively correlated with improved clinical prognosis	67
Colorectal cancer	Low expression	Western blotting, RT-qPCR	10 pairs of rectal adenocarcinoma and adjacent tissue samples	MZB1 inhibits proliferation, migration and invasion of rectal adenocarcinoma cells and promotes apoptosis of rectal adenocarcinoma cells	68,69

## Abbreviations

MZB1: Marginal zone B and B1 cell-specific protein 1
ER: Endoplasmic reticulum
SLE: Systemic lupus erythematosus
RA: Rheumatoid arthritis
IFN-α: Interferon-alpha
TLR: Toll-like receptor
GRP94: Glucose-regulated protein 94
BiP: Binding immunoglobulin protein
pDC: Plasmacytoid dendritic cell
IgA: Immunoglobulin A
IgM: Immunoglobulin M
MM: Multiple myeloma
HCC: Hepatocellular carcinoma
GC: Gastric cancer
BC: Breast cancer
DLBCL: Diffuse large B-cell lymphoma
FL: Follicular lymphoma
CLL: Chronic lymphocytic leukemia
NSCLC: Non-small cell lung cancer
UPR: Unfolded protein response
